# Uniform distribution of three *Candida albicans *microsatellite markers in two French ICU populations supports a lack of nosocomial cross-contamination

**DOI:** 10.1186/1471-2334-6-162

**Published:** 2006-11-13

**Authors:** Odile Eloy, Stéphanie Marque, Françoise Botterel, François Stephan, Jean-Marc Costa, Virginie Lasserre, Stéphane Bretagne

**Affiliations:** 1 Laboratoire de Parasitologie, Hôpital André Mignot, Le Chesnay, France; 2 Laboratoire de Parasitologie-Mycologie, Hôpital Henri Mondor-APHP, Créteil, France; 3 Unité Mixte de Recherche BIPAR 956, Université Paris 12, Créteil, France; 4 Service d'Anesthésie-Réanimation, CHU de Pointe-à-Pitre, Guadeloupe, France; 5 Laboratoire de Biologie Moléculaire, Hôpital américain, Neuilly/Seine, France; 6 Université de Pharmacie de Paris 5, Paris, France; 7 Centre National de Référence de Mycologie et des Antifongiques, Institut Pasteur, Paris, France

## Abstract

**Background:**

The nosocomial acquisition of *Candida albicans *is a growing concern in intensive care units (ICUs) and understanding the route of contamination is relevant for infection control guidelines.

**Methods:**

To analyze whether there is a specific ecology for any given hospital, we genotyped *C. albicans *isolates of the ICU of Versailles hospital (Hospital A) and compared the results with those previously obtained in another ICU in Henri Mondor hospital (Hospital B) using three polymorphic microsatellite markers (PMM).

**Results:**

Among 36 patients with at least one positive culture for *C. albicans*, 26 had a specific multilocus genotype, two shared a common multilocus genotype, and 8 had the most common multilocus genotype found in the general population. The time interval between periods of hospitalization between patients with common genotypes differed by 13 to 78 days, thus supporting a lack of direct contamination. To confirm this hypothesis, the multilocus genotypic distributions of the three PMM were compared between the two hospitals. No statistically significant difference was observed. Multiple correspondences analysis did not indicate the association of a multilocus genotypic distribution with any given hospital.

**Conclusion:**

The present epidemiological study supports the conclusions that each patient harbours his/her own isolate, and that nosocomial transmission is not common in any given ICU. This study also supports the usefulness and practicability of PMM for studying the epidemiology of *C. albicans*.

## Background

*Candida *infections are a growing concern in patients hospitalized in intensive care units (ICUs) [1]. Among the yeasts involved, *C. albicans *is still predominant in ICUs, accounting for 55% of yeast bloodstream infections [2,3]. Most of these infections are nosocomial, which raises the issue of their prevention. Understanding the route of contamination is of utmost importance in order to implement adequate preventive guidelines.

Genotyping is an approach that can be used to detect cross-contamination. Several genotyping techniques have been reported [4]. Among them, polymorphic microsatellite markers (PMM) have a high discriminatory power and a high throughput when fluorogenic primers and an automated sequencer are used for analysis[5,6]. The data are computerizable and can be compared.

In a previous study on an ICU [7], we used genotyping to show that cross-contamination with *C. albicans *was unlikely and that most of the patients were colonized with their own strain. The aim of the present study was to determine whether this finding was specific or could be generalized to another ICU in another hospital. Moreover, if a nosocomial acquisition occurs in a given hospital, a *C. albicans *population specific to that hospital is expected. Therefore, we studied the genotypes of *C. albicans *isolates of a different ICU from a new hospital and compared the results with those previously reported to detect potential hospital-specific populations. The genotyping was performed using three PMM known to have a discriminatory power of 0.97 [5].

## Methods

### Study populations

The study was conducted in the ICU of Versailles hospital, referred to as Hospital A, located southwest of Paris, and the results were compared to those obtained in Henri Mondor hospital, referred to as Hospital B, located southeast of Paris, forty kilometres from Hospital A.

The ICU of Hospital A (18-bed ward) treats both medical and surgical patients whereas the ICU of Hospital B (16-bed ward) has only surgical patients. Patients above 18 years of age and at high risk for *Candida *infection were eligible for the study. The criteria for inclusion were recent abdominal surgery (<24 hours), gastrointestinal perforation or anastomotic leakages, urologic tract surgery and/or broad-spectrum therapy for more than eight days [7]. The following information was recorded for each patient: age, sex, new Simplified Acute Physiology Score (SAPS II) [8], presence of diabetes, immunodepression, wide-spectrum antibiotic therapy, orally administered amphotericin B, presence of arterial or central venous catheter, transfusion requirement, total parenteral nutrition, type of surgery and length of ICU stay. The Comité Consultatif de Protection des Personnes dans la Recherche Biomédicale of each hospital confirmed that no ethical approval was required since this observational study did not modify current diagnostic or therapeutic strategies. Written or oral informed consent was obtained from patients or from their relatives.

### Sampling

In both hospitals, patients were sampled for colonization at entry in the ICU and then once weekly. The sampling consisted of skin and mouth swabs, urine, stools and tracheal aspirations. Clinical specimens were cultured on chromogenic medium for 48 hours at 37°C. All green colonies (CHROMagar^® ^*Candida *for Hospital A) and blue colonies (*Candida *ID^®^, BioMérieux for Hospital B were identified as *C. albicans*. The others colonies were identified using commercially available strips (Api 20C^®^, BioMérieux in Hospital A or ATB ID32C^®^, BioMérieux in Hospital B) together with micromorphological analysis on rice-extract agar (Becton Dickinson). One colony per sample was put into 1 ml of 10% glycerol and frozen at -80°C until PCR analysis.

### Microsatellite analysis

The surface of the *C. albicans *frozen aliquots was scraped with a loop and the material obtained was seeded on Sabouraud medium. After 24 h, one colony was directly suspended in 100 μl of sterile water and boiled to free DNA. After centrifugation for 3 minutes at 2000 × g, 2 μl of the supernatant was used to amplify three polymorphic loci named "CDC3", "EF3" and "HIS3" after the genes they are located closest to. Primers were designed to amplify the microsatellite markers and one primer of each set was 5'-labeled with different dyes as previously described [5]. In Hospital A, the PCR products were run in capillary and read using a 310 automated sequencer (Applied Biosystems). The data were stored and analyzed using the 372 GeneScan software (Applied Biosystems).

The 372 GeneScan software automatically calculates the PCR fragment length according to the internal standard and gives fractional numbers. To check the reproducibility of the technique, we tested the B311 reference strain in ten separate experiments. This strain is heterozygous for the three markers and the length in base pairs (bp) of each allele is known [5]. The standard deviation was +/- 0.20 bp for CDC3, +/- 0.24 for EF3, and +/- 0.42 for HIS3. To assign a specific length to a PCR fragment, we systematically tested the B311 strain in all the PCR runs. All the PCR results were aligned with this reference strain. Therefore, each allele was named according to the length in bp of the amplified fragment after the alignment with the reference strain. When several isolates were available for a given patient, all the profiles were also aligned to check their similarity. When we observed electromorphs with one signal for a given locus, we considered that the isolates were homozygous for this locus [9].

### Comparaison of PMM analysis between the two hospitals

In Hospital A, the PCR products were run in capillary and read using a 370 automated sequencer (Applied Biosystems). For Hospital B [7], we ran the PCR products on a 36-cm long acrylamide urea gel and read the signals using a 377 automated sequencer (Applied Biosystems). After analysis by the 372 GeneScan software (Applied Biosystems), we were obliged to make some corrections because of the different automated sequencers used in each place. The differences observed between the 377 – 310 sequencers for 6 reference strains (Ca 4918, IP1877/89, IP996/69, IP1880/89, H12, B311) were 0.4 nucleotide for CDC3, 2.4 nucleotides for EF3 and one nucleotide for HIS3. Therefore, using the B311 strain ran in each series, we calculated the differences between the length obtained with the 310 sequencer and that calculated with the 377 sequencer and reported in previous publications [5]. We added to each allele of each locus the difference calculated to obtain comparative results. As described above, each profile was compared with the B311 strain profile and the length in base pairs was given according to the known length of the B311 alleles.

### Statistical analysis

Two kinds of statistical analysis were used to compare Hospital A and B's populations: the genotypic differentiation test called the G test and a multiple correspondence analysis (MCA). The G test analyzes the diversity of diploid populations on multi-loci where multi-allelic possibilities exist and tests the null hypothesis that the distribution is identical across populations [10]. A likelihood test of the observations is performed and the result is given by a probability *P *based on a Fischer's test. If *P *> 0.05, the compared populations are considered as similar. This test is suitable for molecular markers such as PMM in diploid populations, with a high level of genetic diversity, when the loci can be considered independent [11]. The data were coded and analyzed using the GenePop software version 3.4 June 2003 [12].

A cluster analysis between the two populations was performed by MCA which is an ordination technique. MCA is a type of Correspondence Analysis (CA) which considerably expands the scope of Principal Components Analysis (PCA). The percentage *inertia *explained by axes takes the place of the percentage variance of PCA. PMM data can be coded by 1 (presence) or 0 (absence) for each of the possible allele. Such coding leads to complete disjunctive data used in the MCA. One byproduct is that the row and column projections in the new space may both be plotted on the same output graphic presentations. For our analysis, only the six first axes were considered for explaining the contingency table variability. Scatter plots were chosen for a graphic representation. They were centered in the first two axes. This analysis was performed using the XLSTAT (version 7.5) software package [13]. A hierarchical classification by a neighbor-joining with the squared Euclidian distances was determined from the six first axes of the MCA.

## Results

In Hospital A, 1424 patients were hospitalized from 1^st ^February 2000 to 31^st ^January 2002, and 60 patients were included. Among them, 56 patients (93%) had at least one sample that was positive for *Candida *sp. For 11 of them, the culture was positive as early as the first day of hospitalization in the ICU. The 45 other patients had positive samples after a minimum of 72 hours. In Hospital B, 303 patients were hospitalized from 1^st ^November 1999 to 31^st ^October 2000, and 94 patients were included. Among them, 43 patients (46%) had at least one positive sample in culture and 36 had positive samples after a minimum of 72 hours. Table 1 lists the demographic data and the main characteristics of the patients in the two hospitals who acquired any *Candida *sp colonization after 72 hours of hospitalization. The percentage of acquisition after 72 h was 80.3% (45/56) in Hospital A and 83.7% (36/43) in Hospital B. The number of positive cultures for *Candida *spp. was 156 out of 333 (47%) specimens recovered in Hospital A. The figure was lower in Hospital B where 167 out of the 1126 (15%) samples were culture positive. Despite this finding, there was no significant difference in the distribution of *Candida *species (data not shown).

**Table 1:** Main characteristics of the patients who acquired any *Candida *sp colonization after 72 hours of hospitalization in Hospital A (45/56, i;e. 80.3%) and in Hospital B (36/43, i.e; 83.7%).

Patient characteristics	Hospital A n = 45 (%)	Hospital B n = 36 (%)	*P*
Age (years) mean ± SD	66.4 ± 15.4	62.6 ± 16.4	0.57
Sex ratio (M/F)	25/20	23/13	0.42
SAPS II score: mean points ± SD	45.5 ± 14	37.5 ± 14.5	0.19
Diabetes	4 (8.9)	6 (16.7)	0.28
Immunodepression	17 (37.8)	9 (25.0)	0.26
Previous administration of antibiotics	43 (95.6)	26 (72.2)	0.25
Oral amphotericin B	0	32 (88.9)	0.001*
Central venous catheter	37 (82.2)	34 (94.4)	0.40
Radial artery catheter	21 (46.7)	31 (86.1)	0.06
Transfusion requirements	17 (37.8)	29 (80.6)	0.03*
Total parenteral nutrition	15 (33.3)	27 (75.0)	0.03*
Abdominal surgery	11 (24.4)	21 (57.9)	0.03*
Liver transplant	0	3 (8.3)	0.10
Length of stay: median days (25th-75th percentiles)	21 (11–53)	31 (14.0–55.5)	0.07

*: statistically significant difference. *P *calculated by a Fisher's exact test.

In Hospital A, 82 isolates from different anatomical sites (Table 2) were genotyped from the 36 patients with at least one positive culture for *C. albicans*. The genotype was identical for a given patient whatever the anatomical site. Only one patient sequentially harboured two different genotypes in the same anatomical site (see below). In Hospital B, 122 *C. albicans *isolates from different anatomical sites (Table 2) from 30 patients had been genotyped [5].

**Table 2:** Source of the 82 *C. albicans *genotyped isolates from 36 patients in Hospital A and the 122 *C albicans *genotyped isolates from 30 patients in Hospital B.

	No (%) of positive specimens				
Clinical sample	Hospital A		Hospital B		*P*

	Number	%	Number	%	

Mouth, skin and superficial surgical wounds	38	46.3	1	0.8	0.001*
Peritoneal fluid, abscess, drain effluent and bile	3	3.6	50	41	0.001*
Stools and gastric secretions	15	18.3	18	14.7	0.350
Urinary tract	11	13.4	15	12.3	0.498
Respiratory tract	15	18.3	34	27.8	0.141
Blood	0	0.0	2	1.6	0.361
Central venous catheter	0	0.0	2	1.6	0.361
Total	82	100.0	122	100.0	

* *P *< 0.05: significant differences. (*P *calculated by a Fisher's exact test).

In Hospital A, 36 patients had at least one positive culture of *C. albicans *and 19 patients had serial *C. albicans *isolates. Of these 19 patients, only one patient had two different genotypes of *C. albicans*. The first genotype was specific for the patient while the second genotype, obtained 8 days later, was a genotype found in other patients. Nevertheless, we kept both genotypes of this patient for further analysis. Among these 36 patients, 26 (72%) had a specific genotype, 8 (22%) shared the multilocus genotype characterized by CDC3: 117–125; EF3: 126–135; HIS3: 162-162 whose the frequency is 17% among 100 unrelated isolates [5], and two other patients (6%) shared another identical genotype (CDC3: 117–125; EF3: 130–131; HIS3: 162-162). None of the 8 patients sharing the most common multilocus genotype stayed in the ICU over the same period of time, the time interval between the dates of hospitalization in the ICU ranging from 28 to 78 days. The stay of the other 2 patients differed by an interval of 13 days. In Hospital B, the most common multilocus genotype had been found in three (10%) out of 30 patients whose stay in the ICU differed by 7 to 96 days.

Therefore, 37 genotypes from 36 different patients in Hospital A and 30 genotypes from 30 different patients in Hospital B were available for comparison. The allelic frequencies were not statistically different for 34 of the 38 different alleles observed. Table 3 reports the multilocus genotypes of the *C. albicans *isolates observed in the two hospitals. With the genotypic differentiation, the pairwise analysis across the two populations suggested that the populations were not significantly different (*P *> 0.05) for the three PMM: CDC3 (*P *= 0.12), EF3 (*P *= 0.25) and HIS3 (*P *= 0.12).

**Table 3:** Multilocus genotypes of the *C. albicans *isolates of the two hospitals studied.

CDC3 locus		EF3 locus		HIS3 locus		number of isolates	
Allele 1	Allele 2	Allele 1	Allele 2	Allele 1	Allele 2	Hospital A (n= 37)	Hospital B (n = 30)

113	117	130	136	150	162	0	3
113	117	130	136	162	162	1	1
113	117	136	136	150	162	1	0
113	117	136	136	162	162	1	0
113	117	136	142	158	158	1	0
117	117	126	135	162	162	0	1
117	117	130	136	150	162	0	1
117	117	130	136	154	194	0	1
117	117	133	140	154	154	0	1
117	117	133	144	154	178	0	1
117	117	133	144	154	186	0	1
117	117	136	136	162	162	1	0
117	117	136	144	154	154	1	0
117	117	137	139	182	182	0	1
117	121	131	130	162	186	1	0
117	121	132	135	154	162	1	0
117	121	133	136	154	154	0	1
117	121	136	139	154	154	1	0
117	125	126	126	162	162	1	0
117	125	126	126	162	162	0	1
117	125	126	135	154	162	1	0
117	125	126	135	154	166	1	0
117	125	126	135	158	158	1	0
117	125	126	135	162	162	8	3
117	125	126	135	170	218	0	1
117	125	126	135	202	210	1	0
117	125	126	135	210	218	0	1
117	125	126	135	218	230	1	0
117	125	130	130	162	162	1	1
117	125	130	144	154	154	0	1
117	125	131	130	162	162	2	0
117	125	131	131	162	162	0	1
117	125	131	131	162	190	0	1
117	125	131	131	166	166	0	2
117	125	133	144	154	182	0	1
117	125	136	139	154	154	1	0
117	129	136	146	154	154	0	1
121	121	136	136	154	154	1	0
121	125	130	144	154	154	1	0
121	125	130	144	154	166	1	0
121	125	144	144	174	174	1	0
121	125	145	150	166	174	1	0
125	125	126	135	162	162	1	0
125	125	130	144	154	154	1	0
125	125	130	144	154	166	1	0
125	125	130	145	154	166	0	1
125	125	131	139	194	198	0	1
125	125	136	139	150	150	1	0
125	129	130	144	154	154	1	0
129	129	136	141	150	162	0	2

**Figure 1:**
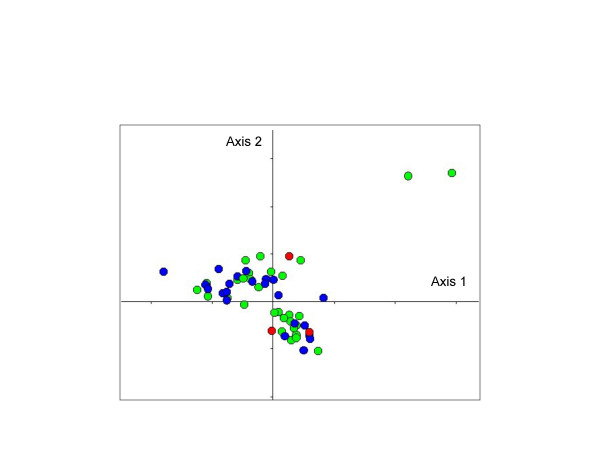
Multiple correspondences analysis for the 37 Hospital A multilocus genotypes and the 30 Hospital B multilocus genotypes. The projection in the plane defined by the two most informative axes is shown (axis 1 variance: 7.56%; axis 2 variance: 7.29%). Although different, some multilocus genotypes are superimposble because of the projection on the plane. Green spots: Hospital A genotypes; blue spots: Hospital B genotypes; red spots: commun genotypes between the two hospitals.

**Figure 2:**
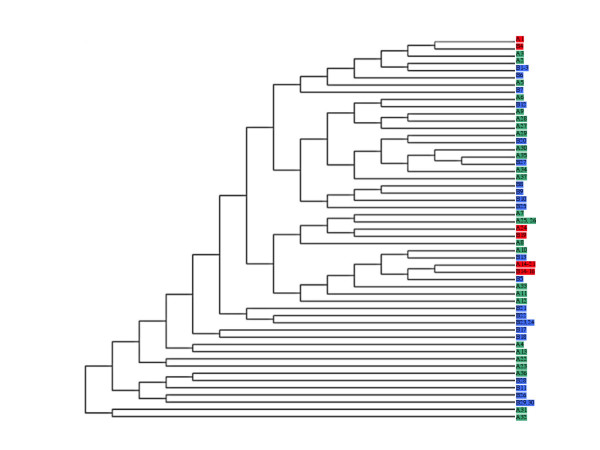
Unrooted tree obtained after hierarchical classification by a neighbor-joining with the squared Euclidian distances determined from the six first axes of the multiple correspondence analysis. Same colour code as in Figure 2. Multilocus genotypes numbers are arbitrary.

The isolates were also analyzed by MCA according to their multilocus genotype. MCA produced a multidimensional representation of the data, which were reduced in plane for convenience. The two first factor axes corresponding to the percentage of the total inertia of 7.56% and 7.29% respectively are shown on Figure 1. Both populations of Hospital A and B are superimposable on this representation without presenting clusters, except for two particular Hospital A multilocus genotypes (A31 and A32 on figure 1B). We determined that only the six first axes (a range of inertia from about 7.6 to 5.1%) present at least one significant correlation (t-test) with the initial variables. To confirm that the two populations were superimposed in all axes, we performed a hierarchical classification by a neighbor-joining with the squared Euclidian distances determined from the six first axes of the MCA. The corresponding unrooted tree is shown on Figure 2. We observed that independently of the origin of the multilocus genotypes, both populations were distributed in all branches of the tree without discriminate some clusters. No association of a genotype with a particular Hospital could be detected.

## Discussion

The aim of the present study was to compare the genotypes of *C. albicans *in two ICUs of two different hospitals to determine whether or not cross-contamination occurs. In Hospital B, a previous study had concluded that there was a low probability of cross contamination between patients [7]. This was confirmed in the present study in Hospital A. Among 36 patients, 26 had a specific *C. albicans *multilocus genotype and, once colonized, the patient usually kept the same genotypes whatever the anatomical site. However, eight patients shared a multilocus genotype which is the most common multilocus genotype among independent isolates [5]. The probability of cross-contamination was low given that they were not hospitalized at the same time in the ICU. Fomites might have had a reservoir role. However, in ICUs, the cleaning procedures between patients exclude this possibility. We cannot rule out that a medical staff member might have served as a common contaminator. However, the most common shared multilocus genotype also belongs to the most common genotype in the general population, which accounted for 17% of 100 unrelated *C albicans *isolates [5]. The fact that some genotypes are more frequent than others has already been reported for *C. albicans *by other authors using PMM [6,14-16]. This may be due to the 0.97 discriminatory power of the present PMM and can be solved by using other PMM [6] to increase the discriminatory power or by using another typing method such as MLST [17]. This may also be due to the lack of a sexual cycle in *C. albicans *and the expansion of some clones [18].

Similarities were found regarding the epidemiology of *C. albicans*, i.e. that most fungal infections that arise in an ICU are caused by unrelated strains, although there were differences between the two hospitals which could have led to less similarities. For instance, the ICU of Hospital A treats medical and surgical patients. In contrast, Hospital B recruits only surgical patients, deemed to be more susceptible to infections since they are more immunocompromised (liver transplant, pancreatitis) or have undergone more invasive procedures. What was in fact surprising was the vast difference in the percentage of positive culture between both hospitals, 47% (156/333) in Hospital A versus 15% (167/1126) in Hospital B. The role of the different media used for fungal growth seems unlikely. An explanation could be could be the role of amphotericin B prophylaxis, implemented in Hospital B only, which could have hampered yeast growth. Other studies have reported similar differences in yeast colonization between 50% [1] and 90% [19]. This underlines the difficulties in standardizing clinical specimens and the microbiological procedures. However, despite these differences, the percentage of patients exhibiting a *C. albicans *colonization after 72 h was similar between the two hospitals (Table 1) and the species distribution was also similar.

The second point of our study was to determine whether there was a *C. albicans *population specific to a given hospital, which might have suggested a nosocomial transmission or a difference in the patient population hospitalized. The G test and the MCA of PMM genotypes concluded with the absence of a *C. albicans *population specific to a given hospital (Figure 1A and 1B). Using the moderately repetitive sequence Ca3 to fingerprint *C. albicans *isolates in two distant hospitals in New Zealand [20], the authors have provided indirect evidence of nosocomial transmission of hospital-specific *C. albicans *strains in one hospital but not the other. Interestingly, these results were obtained under conditions in which no candidiasis outbreak occurred in either hospital, as was the case in our study. Therefore, more systematic screening to investigate *C. albicans *populations in ICUs might suggest intra-hospital acquisition and lead to reinforcement of clinical procedures. Of course, when an outbreak occurs, the genotyping of the *C. albicans *isolates can demonstrate the exogenous acquisition and should lead to preventive measures [21].

The PMM analysis produces computerizable data. However, technical issues should be pointed out since different migration techniques (acrylamide gel versus capillary) and not genome instability led to different allele sizes from the same genotype. The lengths of the PCR fragments and their secondary structures probably interfered with the migration [22]. This should be taken into consideration when comparing results between different laboratories, especially when the lengths of the PCR fragments differ in only one or two bases. Nevertheless, this shortcoming does not change the multilocus genotypes and the discriminatory power does not depend on the migration technique used. Agreement between laboratories should be achieved by using reference strains with known allelic lengths. Despite very few limitations, the PMM technique is well-designed for screening large number of isolates with limited workload. The same technique can be advantageously designed for the study of other yeasts, especially *C. glabrata *[23] which is the second leading species encountered in ICUs.

## Conclusion

The present study reinforces previous statements obtained from a single hospital supporting the fact that patients harbour their own *C. albicans *isolate [7,24]. We also found that there was no genotype specific to any given hospital, thus reinforcing the hypothesis that nosocomial transmission exceptionally occurs in patients hospitalized in the ICU. Therefore, attempts aimed at preventing *C. albicans *colonization should focus on controlling the risk factors rather than on limiting cross-contamination as this yeast rarely appears responsible for cross-contamination between patients in the same ward.

## Competing interests

The author(s) declare that they have no competing interests.

## Authors' contributions

OE, SM, FB, and FS made substantial contribution to acquisition, analysis and interpretation of the clinical and molecular data. J-MC developed and supervised the molecular tests. VL carried out the statistical tests. SB conceived of the study, and drafted the manuscript. All authors read and approved the final manuscript.

## Pre-publication history

The pre-publication history for this paper can be accessed here:



## References

[B1] Eggimann P, Garbino J, Pittet D (2003). Epidemiology of Candida species infections in critically ill non-immunosuppressed patients. Lancet Infect Dis.

[B2] Tortorano AM, Peman J, Bernhardt H, Klingspor L, Kibbler CC, Faure O, Biraghi E, Canton E, Zimmermann K, Seaton S, Grillot R (2004). Epidemiology of candidaemia in Europe: results of 28-month European Confederation of Medical Mycology (ECMM) hospital-based surveillance study. Eur J Clin Microbiol Infect Dis.

[B3] Pfaller MA, Diekema DJ (2002). Role of sentinel surveillance of candidemia: trends in species distribution and antifungal susceptibility. J Clin Microbiol.

[B4] Soll DR (2000). The ins and outs of DNA fingerprinting the infectious fungi. Clin Microbiol Rev.

[B5] Botterel F, Desterke C, Costa C, Bretagne S (2001). Analysis of microsatellite markers of Candida albicans used for rapid typing. J Clin Microbiol.

[B6] Sampaio P, Gusmao L, Correia A, Alves C, Rodrigues AG, Pina-Vaz C, Amorim A, Pais C (2005). New microsatellite multiplex PCR for Candida albicans strain typing reveals microevolutionary changes. J Clin Microbiol.

[B7] Stephan F, Bah MS, Desterke C, Rezaiguia-Delclaux S, Foulet F, Duvaldestin P, Bretagne S (2002). Molecular diversity and routes of colonization of Candida albicans in a surgical intensive care unit, as studied using microsatellite markers. Clin Infect Dis.

[B8] Le Gall JR, Lemeshow S, Saulnier F (1993). A new Simplified Acute Physiology Score (SAPS II) based on a European/North American multicenter study. Jama.

[B9] Costa JM, Eloy O, Botterel F, Janbon G, Bretagne S (2005). Use of microsatellite markers and gene dosage to quantify gene copy numbers in Candida albicans. J Clin Microbiol.

[B10] Raymond M, Rousset F (1995). GENEPOP (version1.2):population genetic software for exact test and ecumenicism. J Heredity.

[B11] Goudet J, Raymond M, de Meeus T, Rousset F (1996). Testing differentiation in diploid populations. Genetics.

[B12] GenePop website. [wbiomedcurtineduau/genepop].

[B13] XLSTAT (version 7.5) software package. [wwwxlstatcom].

[B14] Dalle F, Dumont L, Franco N, Mesmacque D, Caillot D, Bonnin P, Moiroux C, Vagner O, Cuisenier B, Lizard S, Bonnin A (2003). Genotyping of Candida albicans oral strains from healthy individuals by polymorphic microsatellite locus analysis. J Clin Microbiol.

[B15] Lott TJ, Fundyga RE, Brandt ME, Harrison LH, Sofair AN, Hajjeh RA, Warnock DW (2003). Stability of allelic frequencies and distributions of Candida albicans microsatellite loci from U.S. population-based surveillance isolates. J Clin Microbiol.

[B16] Fundyga RE, Lott TJ, Arnold J (2002). Population structure of Candida albicans, a member of the human flora, as determined by microsatellite loci. Infect Genet Evol.

[B17] Tavanti A, Gow NA, Senesi S, Maiden MC, Odds FC (2003). Optimization and validation of multilocus sequence typing for Candida albicans. J Clin Microbiol.

[B18] Lott TJ, Effat MM (2001). Evidence for a more recently evolved clade within a Candida albicans North American population. Microbiology.

[B19] Sandven P, Giercksky KE (2001). Yeast colonization in surgical patients with intra-abdominal perforations. Eur J Clin Microbiol Infect Dis.

[B20] Schmid J, Tay YP, Wan L, Carr M, Parr D, McKinney W (1995). Evidence for nosocomial transmission of Candida albicans obtained by Ca3 fingerprinting. J Clin Microbiol.

[B21] Viviani MA, Cogliati M, Esposto MC, Prigitano A, Tortorano AM (2006). Four-year persistence of a single Candida albicans genotype causing bloodstream infections in a surgical ward proven by multilocus sequence typing. J Clin Microbiol.

[B22] Wenz H, Robertson JM, Menchen S, Oaks F, Demorest DM, Scheibler D, Rosenblum BB, Wike C, Gilbert DA, Efcavitch JW (1998). High-precision genotyping by denaturing capillary electrophoresis. Genome Res.

[B23] Foulet F, Nicolas N, Eloy O, Botterel F, Gantier JC, Costa JM, Bretagne S (2005). Microsatellite marker analysis as a typing system for Candida glabrata. J Clin Microbiol.

[B24] Taylor BN, Harrer T, Pscheidl E, Schweizer A, Rollinghoff M, Schroppel K (2003). Surveillance of nosocomial transmission of Candida albicans in an intensive care unit by DNA fingerprinting. J Hosp Infect.

